# Do muscle synergies reduce the dimensionality of behavior?

**DOI:** 10.3389/fncom.2014.00063

**Published:** 2014-06-23

**Authors:** Naveen Kuppuswamy, Christopher M. Harris

**Affiliations:** ^1^Artificial Intelligence Laboratory, Department of Informatics, University of ZürichZürich, Switzerland; ^2^Centre for Robotics and Neural Systems and Cognition Institute, Plymouth UniversityPlymouth, Devon, UK

**Keywords:** modular motor control, muscle synergies, dimensionality reduction, system balancing, Hankel singular values, optimal motor control

## Abstract

The muscle synergy hypothesis is an archetype of the notion of Dimensionality Reduction (DR) occurring in the central nervous system due to modular organization. Toward validating this hypothesis, it is important to understand if muscle synergies can reduce the state-space dimensionality while maintaining task control. In this paper we present a scheme for investigating this reduction utilizing the temporal muscle synergy formulation. Our approach is based on the observation that constraining the control input to a weighted combination of temporal muscle synergies also constrains the dynamic behavior of a system in a trajectory-specific manner. We compute this constrained reformulation of system dynamics and then use the method of system balancing for quantifying the DR; we term this approach as Trajectory Specific Dimensionality Analysis (TSDA). We then investigate the consequence of minimization of the dimensionality for a given task. These methods are tested in simulations on a linear (tethered mass) and a non-linear (compliant kinematic chain) system. Dimensionality of various reaching trajectories is compared when using idealized temporal synergies. We show that as a consequence of this Minimum Dimensional Control (MDC) model, smooth straight-line Cartesian trajectories with bell-shaped velocity profiles emerged as the optima for the reaching task. We also investigated the effect on dimensionality due to adding via-points to a trajectory. The results indicate that a trajectory and synergy basis specific DR of behavior results from muscle synergy control. The implications of these results for the synergy hypothesis, optimal motor control, motor development, and robotics are discussed.

## 1. Introduction

There is increasingly a consensus that the solution to the *Degree of Freedom (DoF) Problem* of Bernstein (1967) involves some form of Dimensionality Reduction (DR) resulting from modularization, although it is unclear how exactly this occurs. Of the many kinds of modules that have been proposed (Flash and Hochner, 2005), the muscle synergy hypothesis, typified by coordinated activation of groups of muscles, has in recent times emerged as one of the front runners (Alessandro et al., 2013). Spatio-temporal regularities in activation patterns across many muscles that seemingly are task and subject independent is usually cited as evidence for DR in the muscle synergy hypothesis (d'Avella et al., 2003; Hart and Giszter, 2004; Ivanenko et al., 2004; Ting and Macpherson, 2005; Tresch et al., 2006). Nevertheless, a recurring criticism of the hypothesis is its phenomenological nature and difficulty of falsification (Tresch and Jarc, 2009; Kutch and Valero-Cuevas, 2012). One approach toward validating the hypothesis, is to develop a well grounded theoretical understanding of the functionality offered by muscle synergies for neural control.

Although various formulations have been proposed for muscle synergies in literature (Chiovetto et al., 2013), there are some common characteristics to the various models: (1) there is a task-specific recruitment of task-independent modules; (2) the synergies themselves are considered as input-space generators (d'Avella et al., 2003); (3) suggested in some formulations that the number of modules available for recruitment represents a DR of the control input (Ting, 2007; Chiovetto et al., 2013); (4) there is a linearization of the highly non-linear control problem (Alessandro et al., 2012). From a computational viewpoint, each of these features facilitate real-time control and speed up motor learning. However, from a control perspective, modularization could also potentially constrict the functionality of the system. Consequently, investigators have begin to examine the theoretical basis (Berniker et al., 2009; Alessandro et al., 2012) and the feasibility of experimentally extracted synergies for task control (Ting and Macpherson, 2005; Neptune et al., 2009; McKay and Ting, 2012; de Rugy et al., 2013). We propose that this task-space perspective (Alessandro et al., 2013) must be extended to also incorporate the ability of a given set of muscle synergies to reduce behavior dimensionality. Muscle synergies must be evaluated both for task performance and effectiveness as a reduced dimensional controller. In the context of this paper, we denote behavior dimensionality as simply the (apparent) state-space dimensionality of the dynamics of the motor behavior.

The necessity for reducing behavior dimensionality is best seen from the viewpoint of optimal control theory. Observations of a number of regularities in biological movements that are seemingly task-independent have lead to the claim of optimality principles underlying motor control. One the one hand, several investigators have attempted to uncover empirical rules governing motor behaviors such as the *Fitt's* law, 2/3rd power law (Viviani and Flash, 1995), or the bell-shaped velocity profiles of reaching behaviors (Morasso, 1981). Alternately, the so-called complete models (Todorov and Jordan, 1998) have instead suggested that these features are a consequence of minimizing some performance index; several such candidate indices have been proposed, such as energy, force, accuracy, time, peak acceleration, torque changes etc. (Flash and Hogan, 1985; Harris, 1998b; Todorov, 2004). Nevertheless, it is unclear how organisms might autonomously acquire the optimal behavior; i.e., how the neural instantiation of optimality occurs. Developmental motor hypotheses instead suggest that this optimal control is acquired through an ontogenetic learning strategy (Vereijken et al., 1992; Sporns and Edelman, 1993; Ivanchenko and Jacobs, 2003); typically involving some form of progressive exploration of state-space by an organism. There is also evidence for some form of adaptive optimization mechanism underlying motor control learning (Izawa et al., 2008; Wolpert et al., 2011). However, regardless of the actual mechanism underlying motor learning, large state-space dimensionality has a critical impact on the tractability of iteratively acquired optimal behavior, i.e., the *learnability* of the control (Kuppuswamy and Harris, 2013). DR in this case might have a vital role to play in guaranteeing a tractable developmental acquisition of control. We contend that a control strategy composed of synergies, in addition to input dimensionality reduction, must also facilitate a reduction in the dimensionality of the state-space relevant to the optimal motor control problem. This entails an analysis of the DR resulting from the constraints placed on the dynamics due to muscle synergy control.

The reduced dimensional control perspective on muscle synergies was investigated by Berniker et al. (2009), who proposed a time-invariant synergy synthesis technique that utilized the method of system balancing (Lall and Marsden, 2002) for DR of the dynamics. A task variable relevant reduced dimensional dynamic model was generated from an accurate musculo-skeletal model of a frog's leg. This reduced dimensional model was used for synergy synthesis and control planning. Although the method yields synergies that closely correspond with those extracted experimentally, it must be noted that the time-invariant synergy formulation does not conveniently encode the temporal complexity of natural behaviors. For instance, in the analysis of locomotor movements it has been shown that temporal synergies (Ivanenko et al., 2003, 2004, 2005) are more effective in capturing the temporal aspects of the muscle activation patterns at various instances within a gait cycle. Temporal synergies are characterized by a dominant timing sequence that are seemingly independent of sensory feedback (Ivanenko et al., 2005). The synergies can then be interpreted as a pool of task-independent fixed temporal patterns that are selectively recruited in a task-dependent manner for generating the necessary muscle activation (Chiovetto et al., 2013). This formulation has also been used to model motor skill development; an increasing pool of synergies is seemingly employed by adults when compared with infants (Dominici et al., 2011), or in allowing increased behavioral complexity (Ivanenko et al., 2005). Therefore, we use the temporal synergy formulation for exploring the DR in motor behaviors of a system. The control input is composed of a weighted combination of task-independent orthonormal basis patterns as synergies—the weight matrix uniquely specifies the behavior (trajectory) of the system. This enables us to extend the procedure of Berniker et al. (2009) to generate both a task variable, as well as synergy basis relevant analysis of the DR of motor behavior.

In this paper, we first develop a method for the analysis of the constraints placed on the dynamics due to temporal muscle synergy control. For a given dynamical system, where the set of synergies, and the weight matrix corresponding to a given trajectory are pre-specified, a “*constrained reformulation*” of the dynamics is computed. This is a trajectory, and synergy basis specific constrained reformulation of the dynamics where the temporal synergies are treated as control inputs triggered for the duration of the movement. We then quantify the DR by using the approach of system balancing (Moore, 1981; Hahn and Edgar, 2002; Lall and Marsden, 2002). This approach preserves the features of the dynamics that are most relevant to control; the subspace of the state that is most affected by the input (control variables) and in turn has the greatest effect on the output (task variables) is identified.

Our proposed Trajectory Specific Dimensionality Analysis (TSDA) obtains both the dimensionality of this subspace and the corresponding reduced-dimensional dynamics of the system following a given trajectory. We then demonstrate that synergies can contribute to a DR in behaviors, however, the resulting reduction is specific to the synergy basis utilized and the trajectory that is followed in order to realize the task. We test our methods in simulations on two kinds of systems: (1) a linear system composed of a tethered mass, and (2) a non-linear compliant kinematic chain, and contrast the DR in performing reaching tasks in various trajectories. Idealized temporal synergies composed of Legendre polynomial and the Fourier bases are used for the experiments.

We then examine the consequences of reducing the dimensionality of a given task to the greatest extent possible. A cost function for quantifying the dimensionality is developed using the system balancing measure of Hankel Singular Values (HSV). Numerical minimization of this cost function obtains the weight matrix, and the corresponding trajectory, that best minimizes the dimensionality while satisfying the task constraints. This control model of Minimum Dimensional Control (MDC) is tested in the simulated linear and non-linear systems for two kinds of tasks: (1) reaching tasks, (2) via-point tasks. From the results it can be seen that smooth trajectories with bell-shaped velocity profiles emerges as the optima. Furthermore, we show that the velocity profiles of the trajectories are dependent on the temporal synergy basis that is employed. The similarity of the resulting trajectories to experimentally observed human behaviors lead us to hypothesize that a dimensionality reduction principle might underlie motor control.

We introduce our approaches in the following way: In section 2 we first outline the temporal synergy control problem and review dimensionality reduction and system balancing. Subsequently we derive the TSDA and our proposed minimization model of MDC. This is followed by a description of the simulation setup and experiments in section 2.5 and the results in section 3. We then discuss the implications in section 4.

## 2. Materials and methods

We first introduce some basic formalism to the optimal control problem. Consider the following representation of the neuro-mechanical dynamics,

(1) $$y(t)=h(x,t),\;\dot{x}=f(x,t)+g(x,u,t),$$

where the variables **x**(*t*) denotes the state, **u**(*t*) the input, and **y**(*t*) the output. For this system the state-space dimensionality can be described by **x**(*t*) ∈ ℝ^*N*^, the input by **u**(*t*) ∈ ℝ^*N*_*i*_^, the output by **y**(*t*) ∈ ℝ^*N*_*o*_^ and *N*_*i*_ and *N*_*o*_ need not be equal to *N*. We utilize a continuous-time deterministic control system description, so **u** can be considered to lie in the infinite dimensional space of continuous functions. Let us define this system by 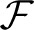
(*f*(·), *g*(·), *h*(·)), where, 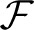 ∈ Ω, a space of sufficiently regular (continuously differentiable) functions.

Although in this paper, we consider **u**(*t*) to be input joint torques or actuator forces, the approach is unaffected if muscle activation dynamics are instead incorporated. The aim of control in the system 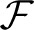 is to influence the behavior in order to satisfy task requirements. For the scope of this paper, we simply define behavior as the trajectory followed by the system in accomplishing a task. A task 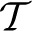 is then denoted by a set of Cartesian constraints that must be obeyed, i.e., by the tuple *C*_
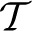
_ = {**y**_
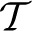
_(*t*_*d*_) = **y**_
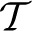
*t*_*d*__, $\dot{x}$_
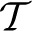
_(*t*_*d*_) = $\dot{x}$_
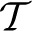
*t*_*d*__}. The constraints are specified by a set of boundary conditions on the behavior such as zero endpoint velocity for reaching, or as a discrete set of via-points to be followed.

A trajectory is then denoted by *T*, one of the many possible unique paths in the task space satisfying all of the task constraints *C*_*T*_. For this system, from an engineering perspective, the feedforward control problem is to compute the function (or policy) **u**(*t*) = *f*_*f*_(,**x**(*t*_0_)). Let us denote then **u**(*t*) ∈ 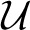 as the set of admissible control inputs that satisfy the desired objectives *C*_
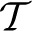
_. There may exist multiple solutions for the task, i.e., multiple trajectories, and therefore the cardinality of 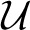 could be considered to be greater than 1. This relation is the well-known *redundancy problem* of motor control, i.e., there is a non-univocal relationship between observed movements and input actuation (Bernstein, 1967).

Many investigators have suggested that the solution to the redundancy problem arises from minimizing some form of cost function *J*(**x**(*t*), **u**(*t*), *t*)—i.e., an underlying optimization principle to motor control. Typically such cost functions have been justified by citing various biologically relevant factors that impact survival such as energy requirements, accuracy, stability of control etc. (Hogan, 1984; Harris and Wolpert, 1998; Todorov and Jordan, 2002).

The optimal control approach to solve this problem typically is based on methods such as solutions to Hamilton-Jacobi-Bellman (HJB) equation or the Pontryagin Minimum Principle (PMP) (Bertsekas, 1995). However, it may not always be possible to obtain analytical solutions to problems—complexity of plant dynamics and the requirement for accurate dynamic models have been major issues. Also, proponents of optimality in biological motor control do not really address how an organism might autonomously acquire optimal solutions. It is instead implied that some form of motor learning or adaptation at different time scales allows the acquisition of optimal behavior (Wolpert et al., 2011). Several developmental theories, such as the Bernstein's three-stage learning model (Bernstein, 1967) have been put forward to how this might be autonomously acquired through a process of state-space exploration. In this context, state-of-art methods in artificial systems such as iterative optimal control and the algorithms of reinforcement learning (Sutton and Barto, 1998) have proved to be a popular alternative to analytical optimal control techniques and have found many applications in areas such as robotics (Kober et al., 2013).

Regardless of the actual mechanism of neural learning, for the system in Equation (1), the complexity of control learning is dictated by a number factors such as the dimensionality of the input *N*_*i*_, the dimensionality of the goal *N*_*o*_, the temporal complexity of the goal trajectory **y**_
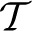
_(*t*_*d*_), the complexity of the cost function *J*(**x**(*t*), **u**(*t*), t) and finally the dimensionality of the state, *N*. For even moderate dimensional systems, this represents a serious limitation on the tractability of computing an appropriate control policy. Also non-linearities in the functions *f*(·), *g*(·), and *h*(·) can further complicate the problem.

Even from a neuroscientific perspective, most investigations in optimal motor control have focussed on relatively simpler models approximating real musculo-skeletal structures (Harris, 1998b). However, optimal control models such as the minimum energy, minimum torque change, minimum jerk, and the minimum variance may instead be intractable for an organism when confronted with anything more than a moderate number of dimensions. Clearly the redundancy and dimensionality problem is not just a motor neuroscience question but represents a constraint on learning for an organism (Kuppuswamy and Harris, 2013). The famous phrase “*curse of dimensionality*” coined by Bellman (1961) to describe the exponential increase in search space of discrete optimization problems due to dimensionality increase seems appropriate in describing this predicament. DR in this case offers an obvious coping strategy wherein the tractability of control learning can be ensured. It has therefore been suggested that neural architectures must intrinsically incorporate some form of DR such as the muscle synergies. The temporal muscle synergy formulation is introduced next within this framework.

### 2.1. Temporal muscle synergy formulation

Most models of the muscle synergy hypothesis tackle the DoF problem by constraining the space of control inputs into combinations of predefined primitives. The temporal synergy formulation has the advantage of conveniently delineating the spatial task-dependent and temporal task-independent components of a synergistic control (Alessandro et al., 2013). Temporal synergies are primarily relevant to locomotor tasks and are a direct example of dimensionality reduction in the control input (Ivanenko et al., 2003, 2004, 2006; Cappellini et al., 2006) with relevance to development and evolutionary theories (Ivanenko et al., 2005; Dominici et al., 2011). Chiovetto et al. (2013) tested the equivalence of temporal muscle synergies with the other main formulations of time-invariant and time-varying synergies on reaching task. The temporal synergy model also has the added advantage of allowing interpretation of the temporal components of the muscle activation occurring at different segments of the movement.

In this formulation, the input **u**(*t*) is constrained in the form of a weighted linear combination of *S* synergies ψ_*i*_(*t*) represented by,

(2) $$u(t)=\sum_{i\;=\;1}^{S}w_{i}\psi_{i}(t),$$

which can be rewritten in matrix notation by *Ŵ*Ψ(*t*) such that Ψ(*t*) = [**ψ**_1_(*t*) … **ψ**_*S*_(*t*)]^*T*^ defines the temporal synergies and the weight matrix *Ŵ* = [*w*_1_ … *w*_*S*_] contains the linear combinators approximating a particular input signal **u**(*t*). In the reported models, arbitrary phase shifts are also included in the synergies, however, we do not incorporate them into the analysis presented in this paper.

There is a unique *Ŵ* for a given **u**(*t*) if the functions **ψ**_1_(*t*), … **ψ**_*s*_(*t*) are linearly independent and *Ŵ* ∈ ℝ^*I* × *S*^, i.e., the synergies are an orthonormal basis set of the space of inputs. The synergies are specified as a task-independent basis spanning the space of inputs, while the appropriate weight matrix is then computed in a task-dependent manner.

**Figure 1:**
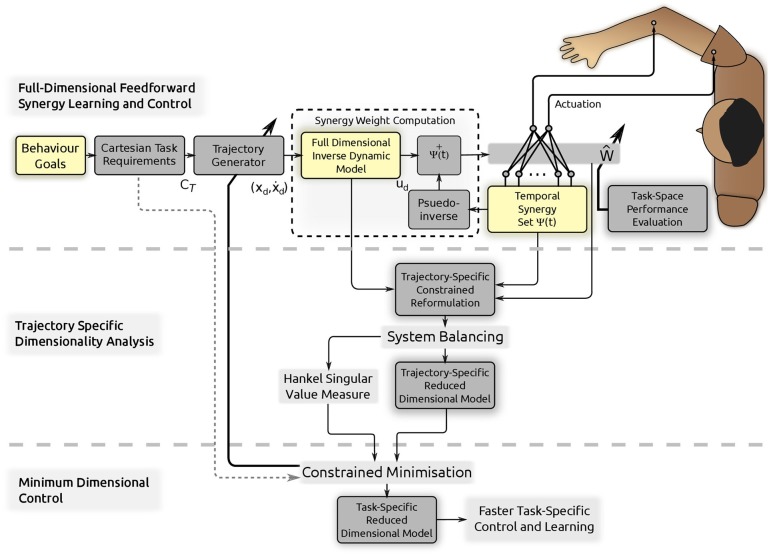
**Conceptual schematic of the proposed methods and control model**. First a simple feedforward control and learning scheme using temporal muscle synergies Ψ(*t*) and full-dimensional system dynamics is used to convert Cartesian task requirements *C*_
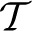
_ obtained from behavior goals into the necessary synergy weight matrix *Ŵ*. A trajectory (and synergy basis) specific constrained reformulation is then obtained and the procedure of system balancing is used to reduce the dimensionality: the proposed Trajectory Specific Dimensionality Analysis (TSDA). The Hankel Singular Value measure computed through system balancing is developed into a performance index for minimization in the Minimum Dimensional Control (MDC) model. The resulting reduced dimensional model can instead be used within the synergy control learning in the control and learning scheme (on top) to speed up learning and adaptation in a task-specific manner.

The control learning problem is to obtain the appropriate weight matrix *Ŵ*_*d*_ corresponding to a desired task **y**_*d*_(*t*). Due to the reduction in dimensionality, the desired solution is within a space of size *N*_*i*_ × *S*. This is a linear space of inputs and therefore learning can be accomplished by a number of tools and superposition can be utilized to generalize to novel problems. The direct approach for trajectory learning in temporal synergies using an inverse dynamic model can be seen in the top part of the schematic in Figure 1.

Despite the reduction in dimensionality of inputs, we contend that the complexity of the optimal motor control problem may not necessarily be reduced simply through reduction of input space dimensionality. For instance, if the desired cost function is a function of the state **x**, the state dimensionality is a bottleneck affecting learnability. Also, the specification of the task might have an important role to play in existing methods of quantifying the dimensionality of synergies (de Rugy et al., 2013); i.e., the number of synergies may be insufficient to ensure optimal control or learning convergence.

In the case of synthetic systems, parameterized control policies in this form (sometimes also called as motor primitives Ijspeert et al., 2013) have been successfully applied in planning and control for robotics. Reinforcement learning approaches such as policy gradients (Peters and Schaal, 2008) offer several methods for iteratively updating policy parameters depending on some predefined behavior objective. However, in the synthetic context, several *a priori* design choices must be carefully made in order to ensure convergence of the learning within reasonable time-scales in high-dimensional control problems (Kober et al., 2013); DR is one such approach toward rendering optimal control learning tractable. Clearly, if the policy design itself could facilitate DR of a system for a given task, the learning would in turn be naturally facilitated.

The primary question investigated in this paper is therefore: do temporal muscle synergies reduce the state-space dimensionality of the system in performing motor behaviors? Next, dimensionality reduction and system balancing are briefly introduced.

### 2.2. Dimensionality reduction and Hankel singular values

From the control engineering viewpoint, the aim of dimensionality reduction is to simplify the input–output dynamics of a system in order to reduce the complexity of simulation and control optimization. Many algorithms have been proposed for model and controller order reduction (Antoulas et al., 2001) including both analytic and computational methods. Consider the state-space model of a system in Equation (1). The DR problem is the synthesis of an equivalent system given by,

(3) $$\tilde{y}(t)=h^{\prime }(z,\;t),\;\dot{z}\;=\;f^{\prime }(z,\;t)\;+\;g^{\prime }\;(z,\;u,\;t),$$

where **z**(*t*) ∈ ℝ^
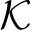
^, and typically the dimensionality of the new state variable 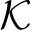 < *N*. Note that when driven by input signals **u**(*t*) the output of the reduced system is **ỹ**(*t*) is close to **y**(*t*) for some measure of similarity. The dimensionality of the inputs and outputs remain unaffected by the reduction.

We seek a quantification of DR in a system instead of simply reducing it to the form of Equation (3). Therefore, we define the reduced dimensionality of a system by the operator 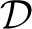, 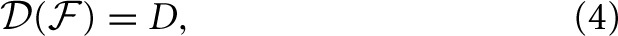 where *D* ∈ ℤ^+^, the space of positive integers. For the system defined in Equation (1), 1 ≤ *D* ≤ *N* for any given measure of dimensionality, or reduction algorithm. Obviously, *D* = 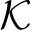 for the reduction leading to the system in Equation (3).

In order to achieve this kind of a reduction, the commonly used approach is to compute a projection of the full dimensional state into a lower dimensional subspace. This is defined as a mapping *W*, such that, **z** = *W***x**. Various methods exist for computation of an appropriate *W*, such that certain conditions are met in the input, state and output relationship. We utilize the well known method of system balancing (Moore, 1981) due to its relevance for control and stable numerical properties. System balancing also offers bounds on the approximation errors (Gugercin and Antoulas, 2004) which is crucial for robust controller design.

Through system balancing, we seek to rotate the system coordinates (i.e., the state-space) in order to balance the controllability (difficulty of reaching a state) and observability (difficulty of observing a state) of the system (Skogestad and Postlethwaite, 1996). This process reorganizes the system by ranking the importance of each of the state variables using a Hankel Singular Value (HSV) measure. They are defined as the square root of the eigenvalues of the product of the controllability (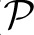) and observability Gramians (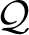); measures computed on the dynamics of the system. For a Linear Time Invariant (LTI) system in the form of Equation (1), defined by the matrices in *f*(**x**, *t*) = *A***x**(*t*), *g*(**x**, **u**, *t*) = *B***u**(*t*), and *h*(**x**, *t*) = *C***x**(*t*), analytical formulations exist for the Gramians defined by, 
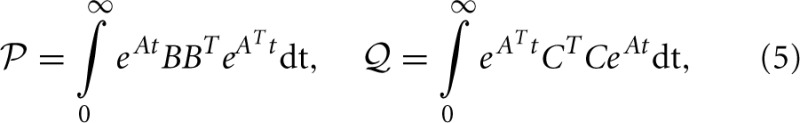
 For non-linear systems, there is no analytical solution but instead *Empirical Gramians* may be computed using datasets of system behavior (Lall and Marsden, 2002).

First the system is perturbed in *r* different (input) directions (defined by the set *T*^*n*_*i*_^ = {*T*_1_, …, *T*_*r*_}, where *T*^*T*^_*i*_*T*_*i*_ = *I*, *T*_*i*_ ∈ 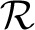
^*n*_*i*_ × *n*_*i*_^, *i* = 1 … *r*) at *s* different sizes of perturbations in each direction (defined by the set *M* = {*c*_1_, …, *c*_*s*_} where *c*_*i*_ ∈ 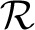, *c*_*i*_ > 0, *i* = 1 … *s*) across all the *n*_*i*_ inputs and across all *n* states (defined by the set of unit vectors *E*^*n*^ = {**e**_*i*_, …, **e**_*n*_}) of the system. Then the empirical Gramians are obtained from the resulting state trajectories as, 
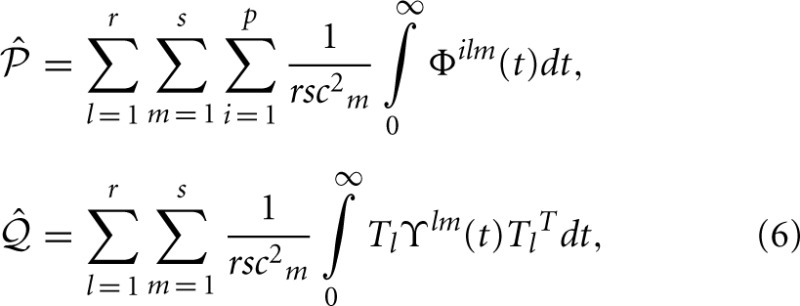
 where for the controllability Gramian 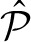, Φ^*ilm*^(*t*) ∈ 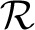
^*n* × *n*^ is given by Φ^*ilm*^(*t*) = (**x**^*ilm*^(*t*) − **x**_0_^*ilm*^)^*T*^, for **x**^*ilm*^(*t*) being the state of the non-linear system corresponding to the impulse input **u**(*t*) = *c*_*m*_*T*_*l*_**e**_*i*_δ(*t*) and for the observability Gramian 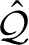, ϒ^*ilm*^_*ij*_(*t*) ∈ 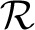
^*n* × *n*^ is given by ϒ^*ilm*^_*ij*_(*t*) = (**y**^*ilm*^(*t*) − **y**^*ilm*^_0_)^*T*^(**y**^*ilm*^(*t*) − **y**^*ilm*^_0_), and **y**^*ilm*^(*t*) is the output of the system for the initial condition **x**(0) = *c*_*m*_*T*_*l*_**e**_*i*_ + **x**_0_, and **y**^*ilm*^_0_ is the steady state output. A detailed description of the non-linear balancing model reduction utilizing the empirical Gramian method can be found in Hahn and Edgar (2002).

These Gramians allow quantification of how controllable and how observable the state variables are; taken together they measure the “importance” of individual state variables and can thus be used for a dimensionality reduction algorithm. For both linear and non-linear systems, the Hankel Singular Values (HSV) of a system σ_*HSV*_ are then obtained as, 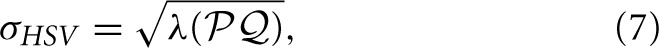 where the λ operator yields the eigenvalues of the product matrix, and the resulting set σ_*HSV*_ = [σ_1_ … σ_*N*_] are the HSVs corresponding to each state variable.

The HSVs can be viewed as a score of the control ‘energy’ of the state variables. Thus to reduce dimensionality it is sufficient to eliminate the states with a low HSV magnitude. This process can be automated by first obtaining a rotation on the system *T* of the form $\hat{x}$ = *T***x** which reorders the states in decreasing magnitude of HSV—i.e., system balancing. This results in a transformation of the system to a basis where the transformed states that are easiest to reach (control) are simultaneously easiest to measure (observe). Computational efficient methods exist for linear systems for computing the balancing transform *T* (Laub et al., 1987). Then its possible to truncate the resulting system to the first 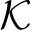 states—hence the method is called balancing truncation (Moore, 1981). The choice of 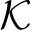 is typically dependent on the requirements of the controller design and is usually fixed after examination of the HSV magnitudes (Hahn and Edgar, 2002).

If the HSVs are normalized by using the sum, the DR is directly given by, 
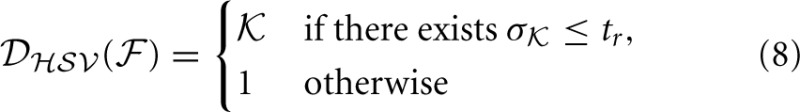
 where the threshold *t*_*r*_ ∈ ℝ^+^, *t*_*r*_ ≤ 1, and the resulting 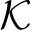 ∈ ℤ^+^, with 1 < 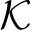 ≤ *N*. Clearly, this form of DR is dependent on the choice of threshold. In the case of control engineering applications, the threshold is chosen on the basis of careful observation of the system (Antoulas et al., 2001). In our approach, presented next, we present a method to simplify choice of this threshold.

### 2.3. Trajectory specific dimensionality analysis (TSDA)

Through system balancing we can quantify the DR of a system. This is a task-independent quantification and depends on the system properties, for e.g., the passive mechanical properties. However, if DR is to be utilized in order to facilitate learning and real-time control of various tasks, the task-dependent reduction of the state-space must instead be considered.

Figure 1 depicts the stages of TSDA computation. The first step is to evaluate the constraints on the system dynamics resulting from the constraints placed on the input due to usage of temporal muscle synergies. The system in Equation (1) can now be represented by,

(9) $$y(t)=h(x,\;t),\;\dot{x}=f(x,\;t)\;+\;\hat{g}\;(x,\Psi ,\;t),$$

We term this as a constrained reformulation of the system dynamics where the inputs are the temporal synergies Ψ(*t*), and can be viewed as signals which control the onset and termination of the movements for a task. For the duration of the behavior, the dynamics is described by Equation (9) due to the constrained input function *ĝ*(·) where,

(10) $$\hat{g}\;(x,\Psi ,\;t)=g\;(x,\;\hat{W}\Psi ,\;t).$$

It must be emphasized that the constrained reformulation only describes a *virtual* system dynamics for the duration of the movement when actuated by the synergistic input Ψ(*t*). The state-space, however, has not changed; i.e., the state variable *x* for constrained-reformulated system is the same as the original system. Let us denote the system of Equation (9) by 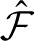
(*f*(·), *ĝ*(·), *h*(·)).

Clearly, $\hat{F}$ is unique to a given trajectory and given synergy basis set, since it incorporates the weight matrix *Ŵ* corresponding to a trajectory *T* and uses input signals in the form of temporal synergies. Therefore 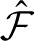 can be considered to be a trajectory specific constrained reformulation of the dynamics. Then the trajectory specific dimensionality is given by, 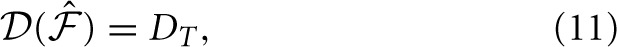 If *Ŵ* is computed to solve a given task 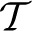 uniquely, Equation (11) gives the DR of the equivalent trajectory that satisfies the task requirements. The TSDA measure can be contrasted against the intrinsic DR of the system of Equation (4), which is task independent.

In this formulation, although any kind of DR algorithm can be utilized for computing *D*_
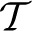
_, we use the system balancing and HSV based approach due to its relevance for the control problem. HSVs measure the importance of each of the state variables of the system 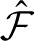 for both the outputs (the task) and the inputs (synergy patterns). Thus they quantify the DR of the behaviors that is dependent on the kind of synergy used and the kind of task that is being performed.

In order to compute the DR, it is desirable that the importance of the careful choice of the threshold HSV measure of Equation (8) is reduced. Depending on the structure of the constrained-reformulated system, it can be expected that HSVs computed for different trajectories may be of completely different orders of magnitudes. Even if normalization using the sum of the HSVs is employed, this may complicate the choice of threshold to compare trajectories. Furthermore this could limit the applicability of the method in comparing different kinds of temporal synergies in reducing the dimensionality.

In order to address this issue in our approach, we simply normalize the HSVs after utilizing a cumulative sum. First the individual HSVs are redefined by,

(12) $${\tilde{\sigma }}_{i}=\sum_{j\;=\;1}^{i}\sigma_{j}/\sum_{l\;=\;1}^{N}\sigma_{l},$$

therefore, the vector $\tilde{\sigma }$_*HSV*_ is the normalized cumulative sum of σ_*HSV*_. This process renders the relationship $\tilde{\sigma }$_*HSV*_*N*__ = 1. Thus, independent of basis or the weight matrix magnitude, the threshold can be chosen to lie in the interval 0 < *t*_*r*_ < 1. We later discuss the implications of the choice of threshold magnitude on motor skill development.

Using the threshold normalized HSVs, the trajectory specific dimensionality is therefore given by, 
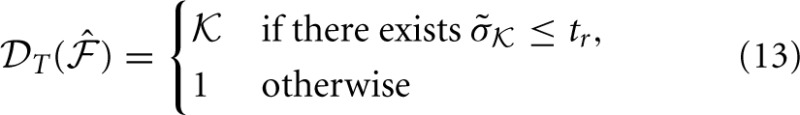
 The TSDA can therefore be computed for both linear and non-linear systems. It must also be noted that through computation of the TSDA, an equivalent trajectory-specific reduced dimensional model of the behavior is also computed as described in Figure 1. We now extend these methods in order to examine the implications of dimensionality minimization, as described next.

### 2.4. Minimum dimensional control (MDC)

The objective of this paper is to test the supposition that temporal muscle synergies lead to a DR of the state-space dimensionality. Through the method presented developed in the previous section, we can compare various trajectories that satisfy task requirements in terms of the reduction in dimensionality. Now we examine the consequence of minimization of this DR for a given task and a given synergy basis. We define the minimization problem as follows.

As described earlier, for an orthonormal basis set of temporal synergies Ψ(*t*) each weight matrix *Ŵ* corresponds to a unique trajectory in state-space (for the same initial conditions of the dynamical system). Therefore the problem is posed as a constrained minimization for identifying the optimal weight matrix Ŵ^*^_
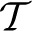
_ that minimizes a dimensionality performance index *J*(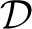
_*T*_) while satisfying task constraints *C*_
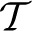
_ as, 
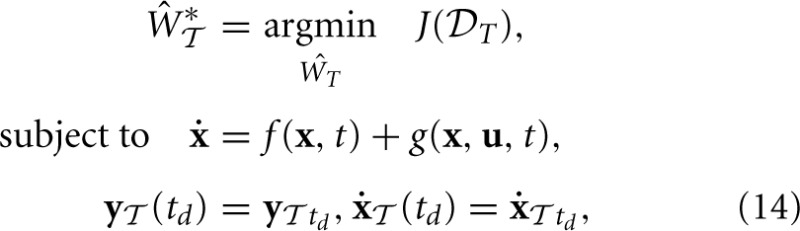
 where the task is specified by a set of task-space and state-space constraints. We term the solution to this minimization problem as Minimum Dimensional Control (MDC) as depicted in Figure 1. The key to this approach is the specification of an appropriate performance index.

In order to generalize our approach to different kinds of physical systems, a computational (numerical) solution is ideally sought. Therefore, the desired properties of this performance index *J*(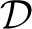
_*T*_) are that it needs to be continuous, and computationally simple for any kind of physical system 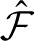.

From the definition of the normalized HSVs in Equation (12), it can be seen that $\tilde{\sigma }$ is a positive, real, bounded, and ordered vector of magnitudes. Also, by definition, the difference between adjacent HSVs, given by δ = $\tilde{\sigma }$_*i* + 1_ − $\tilde{\sigma }$_*i*_, always monotonically decreases toward 0. This implies that the crucial determining factor for minimum reduced dimensionality 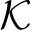 is the magnitude of the second cumulative HSV $\tilde{\sigma }$_2_. This is because the magnitude of subsequent HSVs will be greater, and the first HSV magnitude $\tilde{\sigma }$_1_ is irrelevant for the reduction since *D*_
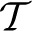
_ ≥ 1.

For any convenient choice of threshold *t*_*r*_, a large magnitude of $\tilde{\sigma }$_2_ ensures that 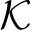 is minimized since all subsequent HSV values ($\tilde{\sigma }$_2_, … $\tilde{\sigma }$_*N*_) are in the interval [$\tilde{\sigma }$_2_, 1]. Effectively, increasing $\tilde{\sigma }$_2_ is equivalent to increasing the range of values of *t*_*r*_ that result in a reduction to a system of subspace dimensionality 1. Clearly, $\tilde{\sigma }$_2_ is the critical magnitude determining reduction in dimensionality.

Based on this rationale the performance index we propose for the MDC is, 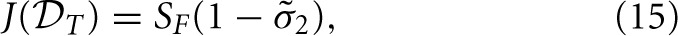 where *S*_*F*_ is a positive rational scale factor. Computationally, the minimization can be carried out using any convenient numerical optimization algorithm. Since the obtained weight matrix Ŵ^*^_
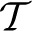
_ is specific to a given task, a given synergy basis set and a given dynamical system, the obtained optimal trajectories are similarly system, task and synergy specific. Despite these conditions, as seen later in the results, invariant characteristics similar to human movements emerge as the optima on the tested linear and non-linear systems. An important consequence of deriving the MDC using the system balancing method is that the approach automatically yields a reduced dimensional dynamic model corresponding to the minimum dimensional trajectory. This is therefore a task-specific reduced dimensional model as depicted in the lower portion of Figure 1.

We hypothesize that the MDC trajectories will lower the difficulty of task learning and optimization. This is particularly relevant for the case of adaptive control, when the dynamics of the system changes with time and optimizing schemes need to keep track of changes, i.e., necessitating a cost on the number of dimensions. The MDC essentially allows task-specific adaptation which can gradually change in a manner mirroring development (Berthier et al., 1999).

It must be noted that MDC itself might be susceptible to the curse of dimensionality and is not meant to explain the neural instantiation of control signals for real-time task planning and control. Instead we propose that it is a model for an optimal mechanism underlying trajectory planning in order to overcome the limitations imposed on the learnability. MDC thus represents a bridge between the muscle synergy hypothesis and the optimal motor control models of redundancy resolution.

### 2.5. Simulation setup

The experiments were performed on two kinds of simulated systems, (1) the linear tethered mass, and (2) a non-linear compliant kinematic chain.

#### 2.5.1. Tethered mass system

**Figure 2:**
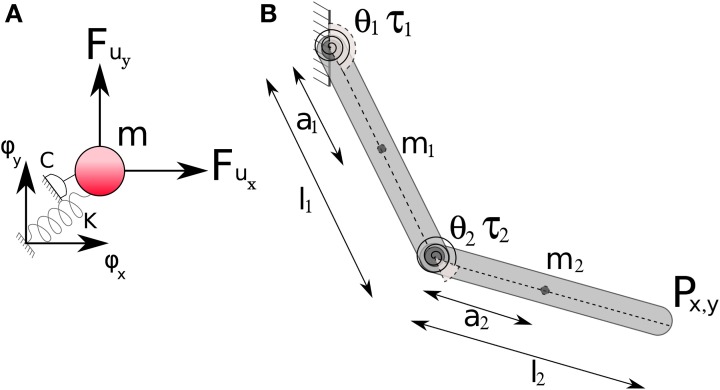
**Physical systems employed for demonstrating the TSDA. (A)** Tethered mass (linear): motion of the mass is constrained to a 2D plane. The mass is anchored to the origin by weak passive forces and actuator forces are applied in two orthogonal directions. **(B)** Two-link planar compliant kinematic chain (non-linear): end-point motion is constrained to a 2*D* surface. Passive joint stiffness and damping effects are present and joint torques are used to actuate the system. The state-space descriptions of these systems have identical input (2), state (4), and output (2) dimensionality.

This system consists of a point mass constrained to move in a 2*D* plane as seen in Figure 2A. It is “tethered” to an origin by weak passive forces using linear springs and is subject to visco-elastic damping. The system can be actuated by independent forces in two orthogonal directions, and the output describes the position in the 2*D* space relative to the origin. The dynamics of this system are described by,

(16) $$\ddot{\phi }=-K\phi \;-\;C\dot{\phi }\;+\;F_{u},$$

where ϕ(*t*) = [ϕ_*x*_(*t*), ϕ_*y*_(*t*)]^*T*^ is the position of the mass in space, *K* is a stiffness matrix, *C* is a damping matrix and **F**_*u*_(*t*) = [**F**_*u*_*x*__, **F**_*u*_*y*__]^*T*^ are orthogonal input forces actuating the system. The simulation parameters were chosen as *C* = 2𝕀 N/m/s and *K* = 6𝕀 N/m.

The system can be considered to be a simplified analog of the oculomotor system. It describes the eye orb dynamics without taking torsional forces into consideration and approximates the passive effects of the orbital tissue. The output can be considered as the displacement angles in horizontal and vertical directions (in radians) since linear approximation of orb movements have been shown to be valid in the range of ±π/6 radians (Bahill et al., 1980).

#### 2.5.2. Compliant kinematic chain

This system is a two-link planar kinematic chain with passive joint compliance as seen in Figure 2B. Actuation is applied through the joint torques. The dynamics are described by Spong and Vidyasagar, (2008),

(17) $$\ddot{\theta }=M{\left(\theta \right)}^{-1}\;[N(\theta ,\;\dot{\theta })\;\dot{\theta }\;+\;K\;(\theta -\theta_{0})\;+\;\tau ],$$

where the state is described by θ(*t*) = [θ_1_(*t*), θ_2_(*t*)]^*T*^, *M*(θ) is denoted the mass-inertia matrix of the system, *N*(θ, $\dot{\theta }$) is the Coriolis matrix and *K* is the joint stiffness matrix and the joint rest positions are given by θ_0_. The system is actuated by the torques τ(*t*) = [τ_1_(*t*), τ_2_(*t*)]^*T*^ at the two joints. The parameters of the simulation are chosen as, *m*_1_ = 0.75 kg, *m*_2_ = 0.5 kg, *l*_1_ = 0.4 m, *l*_2_ = 0.4 m. The applied torques are scaled by a factor of 1.88 at joint 1 and 0.45 at joint 2. A joint stiffness of 0.6 Nm/rad and viscous joint friction of 0.15 Nm/rad is used at both the joints with rest angles fixed at θ(*t*_0_) = [−π/16, π/8]^*T*^. The output of the system is the position **P** = [*P*_*x*_(*t*), *P*_*y*_(*t*)]^*T*^ in the 2*D* Cartesian space which are related to the joint angles through the forward kinematics.

This system describes the behavior of vertebrate limbs. The passive joint compliance not only adds to the biological realism, but also renders the system stable—this is a necessary condition for empirical balancing.

#### 2.5.3. Synergy bases

Two kinds of idealized temporal synergies composed of orthonormal basis functions are tested: (a) Legendre polynomial basis (Ψ_*l*_(*t*)), and (b) Fourier basis (Ψ_*f*_(*t*)) in order to simplify the weight learning for the analysis; they are well known approximators used for curve fitting. They are represented by,

(18) $$\begin{array}{l} \Psi_{l}(t)=\sum_{i\;=\;0}^{n}a_{i}P_{i}\;((2t-t_{d})/t_{d}),\\ \Psi_{f}(t)\;=a_{0}+\sum_{i\;=\;1}^{n}a_{i}\sin\;(i\omega t)\;+\;b_{i}\cos\;(i\omega t), \end{array}$$

respectively, where *t*_*d*_ is the duration of the movement and the weights are given by *Ŵ*_*l*_ = [*a*_0_, … *a*_*n*_], and *Ŵ*_*f*_ = [*a*_0_, *a*_1_, … *a*_*n*_, *b*_1_, … *b*_*n*_]. The Legendre polynomials were computed using the standard Rodriguez formula. Since the polynomials are defined in [−1, +1], they are time-scaled to accommodate the entire duration of the intended movement.

These synergies have another convenient property that their magnitudes are bounded, i.e., abs(Ψ(*t*)) ≤ 1. This property is essential for non-linear TSDA using empirical balancing since the method involves perturbing the inputs using unit impulse signals (Lall and Marsden, 2002). Since the TSDA treats the synergies as input signals, this insures that a unity input perturbation can be applied.

#### 2.5.4. Simulation framework

The simulation was performed on MATLAB (2012). The equations were integrated using the *ode15s* solver in the ODE package with the settings of absolute tolerance = 5*e*^−2^ and relative tolerance 1*e*^−3^. Model reduction routines developed in Hahn and Edgar (2002), and Sun and Hahn (2005) were used for the non-linear system balancing. The weights *Ŵ* for the TSDA benchmark tasks and the MDC initialization were acquired by using a least-squares method. The numerical optimization of MDC was carried out using the *fmincon* routine, with the *interior point* algorithm (Waltz et al., 2006) for the linear MDC and *active set* (Gill et al., 1981) for the non-linear MDC.

## 3. Results

The results of the experiments on the test systems using TSDA and MDC are presented in this section.

### 3.1. TSDA on the tethered mass

**Figure 3:**
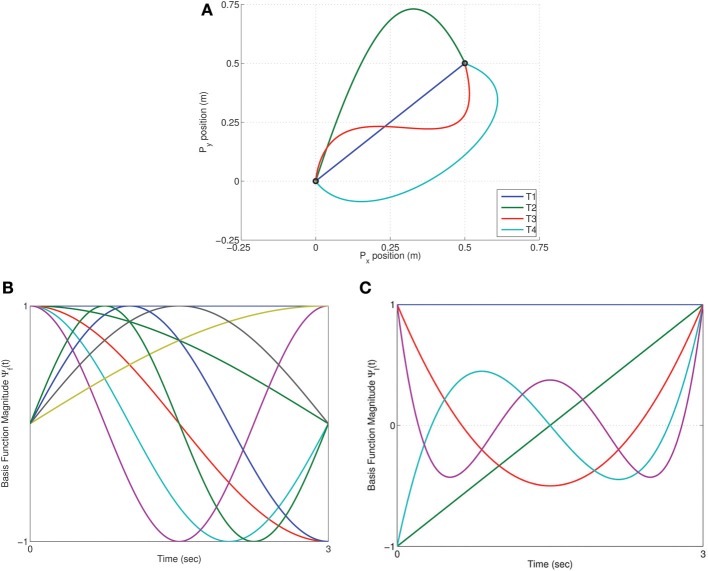
**Trajectory Specific Dimensionality Analysis (TSDA) used to compare four benchmark trajectories. (A)** The task is to reach position (0.5, 0.5) in 3 s tracing each of the four trajectories [*T*_1_, … *T*_4_]. Two kinds of temporal synergies are tested: **(B)** Fourier basis (order 4), and **(C)** Legendre polynomial basis (order 4) actuating the tethered mass system.

A set of four benchmark trajectories, denoted by *T*_*i*_ = ϕ_*i*_(*t*), were compared using TSDA for the tethered mass system. Each trajectory described a motion from the origin to a target output position of [0.5, 0.5], each thus representing a solution to the reaching task. The trajectories, seen in Figure 3A, were specified by via-points in Cartesian space and cubic-spline fit was computed with smoothness conditions enforced at the boundaries (2^nd^ order boundary conditions set to 0). The weight matrix *Ŵ*_*i*_ for the control of each of the trajectories were computed using a least-squares fit of the corresponding inverse dynamic control signals *u*_*d*_*i*__(*t*). Two kinds of synergies were compared: Fourier and Legendre polynomial bases of order 4 each as seen in Figures 3B,C. In the case of the Fourier basis temporal synergy 9 components are necessary corresponding to the sinusoidal and co-sinusoidal parts of the Fourier basis as seen in Figure 3B.

**Figure 4:**
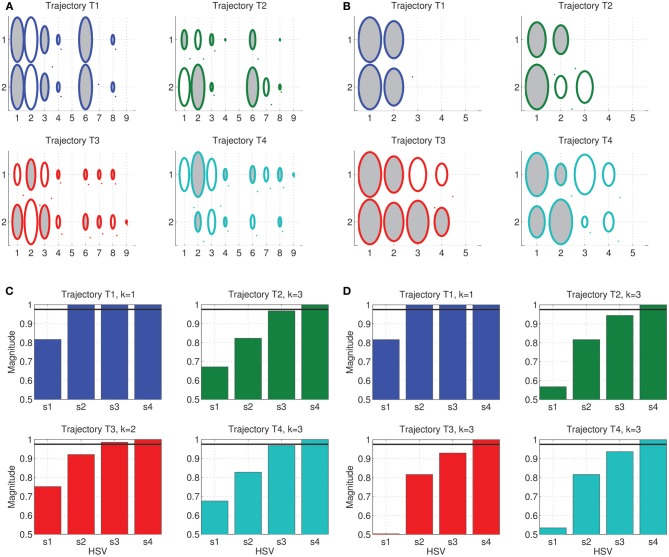
**Trajectory Specific Dimensionality Analysis (TSDA) for comparing the Fourier and Legendre polynomial basis temporal synergies actuating the tethered mass system, tracing the benchmark trajectories [*T*_1_, … *T*_4_]**. The synergy training is carried out using least-squares and full-dimensional inverse dynamics—The obtained weight matrices for the four trajectories are represented as Hinton diagrams (ellipse size is the magnitude, a dark region denotes positive weight and white region denotes a negative weight) for the **(A)** Fourier basis of size 2 × 9, and **(B)** Legendre polynomials of size 2 × 5. The corresponding cumulative normalized HSV magnitudes for **(C)** Fourier, and **(D)** Legendre polynomial basis synergies with the threshold *t*_*r*_ = 0.975 represented in both cases by the solid black line. The DR was computed as 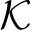
_*fourier*_ = [1, 3, 2, 3], and 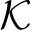
_*legendre*_ = [1, 3, 3, 3]. The straight line trajectory has the minimum dimensionality for both of these synergy bases.

The result of the weight training can be seen in the Hinton diagrams of the weight matrices in Figures 4A,B. The weights, represented by the size of the shaded ellipses, clearly capture the temporal components of each of the trajectories. However, some trajectories are easier to interpret and understand for one kind of synergy alone. For instance, while the weights corresponding to trajectory *T*_1_ are identical in both rows, in the case of *T*_3_, mirroring of weights across the inputs is seen only for the Fourier basis synergy.

For each trajectory, the constrained-reformulated system was constructed and the corresponding reduction, denoted by the vector 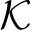
_*T*_, was computed using the linear system balancing procedure. The cumulative normalized HSVs of the constrained-reformulated system can be seen in Figures 4C,D. As noted earlier, the final HSV ($\tilde{\sigma }$_4_ = 1) for all trajectories, i.e., the last bar in each plot is always unity in magnitude. The magnitude of the other HSVs reflect the task, trajectory and the synergy choice.

For this experiment, a threshold value of *t*_*r*_ = 0.975 was utilized to compute the DR (black solid lines in Figures 4C,D). It can be seen that the straight line Cartesian trajectory seemingly has the minimum dimensionality of 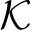 = 1 independent of the choice of threshold magnitude. For the chosen threshold, the DR for each of the trajectories was then obtained as 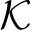
_*fourier*_ = [1, 3, 2, 3], and 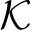
_*legendre*_ = [1, 3, 3, 3]. In the case of TSDA on the Legendre polynomial basis, it can be seen that tasks *T*_2_, *T*_3_, and *T*_4_ are nearly identical in the HSV magnitudes barring minor differences in the 3rd HSV.

The obtained dimensionality on the straight line trajectories imply that it could be a possible candidate for the minimum dimensional solution to the reaching tasks. This is investigated using the MDC framework as described next.

### 3.2. MDC on the tethered mass

In this experiment, the MDC was synthesized for the tethered mass system for a point-to-point reaching task, i.e., with zero velocity at the boundaries. The constrained numerical optimization computed the weight matrix for the synergies which minimize the cost in Equation (15).

For the optimization the initial weights were set using a cubic-spline interpolate of a trajectory fitting the boundary constraints (ϕ(*t*_*d*_) = [0.5, 0.5]^*T*^, $\dot{\phi }$(*t*_*d*_) = [0, 0]^*T*^). A constraint tolerance of ϵ = 10^−2^ was used as a terminal criterion for the minimization. In each of the cases, a local minimum was achieved when using the interior-point algorithm for minimization.

**Figure 5:**
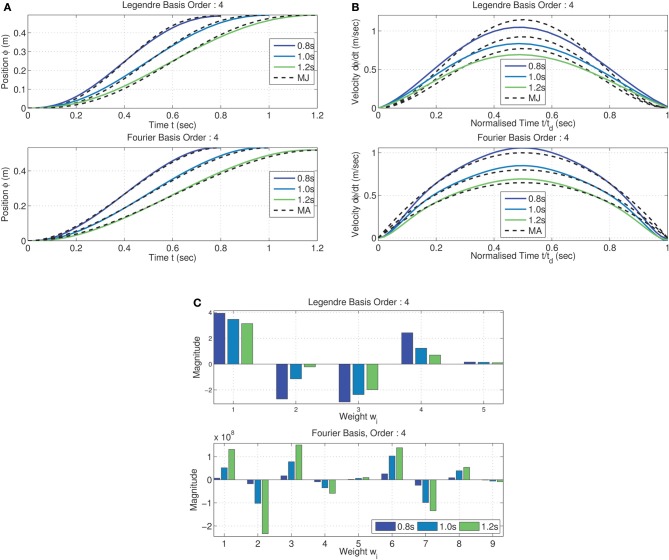
**Minimum dimensional control computed on the tethered mass for reaching position (0.5, 0.5) from the origin—two kinds of synergies (Legendre basis of order 6 and Fourier basis of order 4) and three desired time spans (*t*_*d*_ = [0.8, 1.0, 1.2]) analyzed**. Trajectory of mass traces a sigmoid for all time spans and for both kinds of synergies. Trajectories **(A)** are similar to the minimum jerk (MJ) criterion for the Legendre polynomial basis and minimum acceleration (MA) for the Fourier basis case; **(B)** The corresponding bell-shaped velocity profiles. Weights corresponding to minimum dimension **(C)** for both Legendre polynomial and Fourier basis synergies linearly increase with movement duration across both inputs.

The trajectories resulting from MDC can be seen in Figure 5 for the Legendre, and Fourier basis synergies. Smooth sigmoidal trajectories were obtained as the optimal reaching solution in both cases for multiple movement durations. The terminal cost of optimization was obtained as 2^nd^ HSV $\tilde{\sigma }$_2_ ≈ 0 for all cases. The time normalized velocity profiles, as seen in Figure 5B, are bell-shaped.

Interestingly, from the peak velocities in Figure 5B, it can be seen that while the Legendre polynomial synergies correspond closely to the minimum jerk criterion (Hogan, 1984), the Fourier basis synergy result was a close match with the minimum acceleration criterion (Ben-Itzhak and Karniel, 2008) (represented by the dashed black lines in both cases). There were other minor differences between the trajectories for each kind of synergy. Nevertheless, in both cases the peak velocity of the trajectory scales linearly with the movement duration. The results show that the MDC model computes a synergy specific minimum dimensional trajectory for a given task. It must, however, be noted that MDC does not guarantee symmetric bell-shaped velocity profiles, this is a consequence of the boundary conditions specified and the initialization of the weights for the constrained minimization. Nevertheless, it can be seen that the minimum dimensional solution for the reaching task corresponds to a reduction to a 1 dimensional system independent of the synergy basis chosen.

Due to the linearity of the system, the weight matrix computed by MDC linearly scales with the movement duration as seen in Figure 5C (represented only for one of the inputs). The magnitude of the changes are synergy dependent. This implies that for linear systems the peak velocity and movement duration are a linear function of the synergy weights; the relationship depending on the synergy type.

The tethered mass system can be seen as an analog of the human eye mechanism. The passive forces acting on the mass are similar to the weak passive forces due to the orbital tissue. Although the notion of synergies does not seem to extend to the oculomotor system, the Fourier basis synergy can be viewed as a useful modeling tool for analysis of the frequency response characteristics (Harris, 1998a).

**Figure 6:**
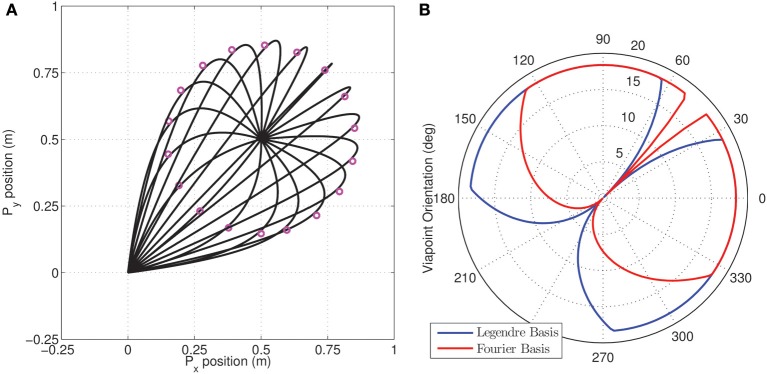
**Dimensionality analysis of via-point tasks. (A)** A set of via-points were specified on a circle of radius 0.353 m centered on the target (0.5, 0.5) for a reaching movement from the initial position (origin); **(B)** Polar plot of the variation in the dimensionality performance index against orientation of via-point with respect to origin for the two kinds of tested synergies composed of Legendre polynomial (blue) and Fourier bases (red). The minimum value of 0 is located exactly along the straight line linking origin and target for both kinds of synergies.

We then used the MDC framework to analyze the reduction in dimensionality in via-point tasks. Via-points were chosen to lie on a circle about the target position (as seen in Figure 6). The via-points were specified to be reached at exactly half the movement duration. In each case, the appropriate synergy weight matrix was computed for both the tested synergy types using an inverse dynamic model and the linear least-squares procedure. In this experiment, instead of just minimizing the performance index, the variation with via-point orientation was obtained, as seen in the polar plot in Figure 6B.

As expected from the earlier reaching experiments, the minimum dimensional via-point is seen to lie exactly along the diagonal, i.e., along the straight line connecting the origin to the target of the movement. Interestingly, for the linear system a minimum dimensional solution was also obtained for the via-points corresponding to the reversal task, i.e., the via-points that lie beyond the target position but along the same line connecting origin and target. Reversal tasks and straight-line reaching are therefore seemingly identical in dimensionality for the linear system. This result also implies that the symmetry of velocity profiles is not guaranteed through MDC, rather it is a consequence of the boundary conditions utilized.

In general, however, the results indicate that for the tested linear system, the choice of via-point can strongly impact the dimensionality of the dynamics. Furthermore the synergy basis specific nature of the dimensionality in following via-points can be seen in the difference between the blue (Legendre polynomials) and red (Fourier basis) lines in Figure 6B. Clearly, the differences in performance index with orientation between the two synergies indicate that certain via-points are ‘easier’ to reach with one kind of synergy basis. This observation is an ideal test-scenario for experimental investigation with subjects and could potentially be used to identify the most appropriate experimentally extracted synergy basis.

**Figure 7:**
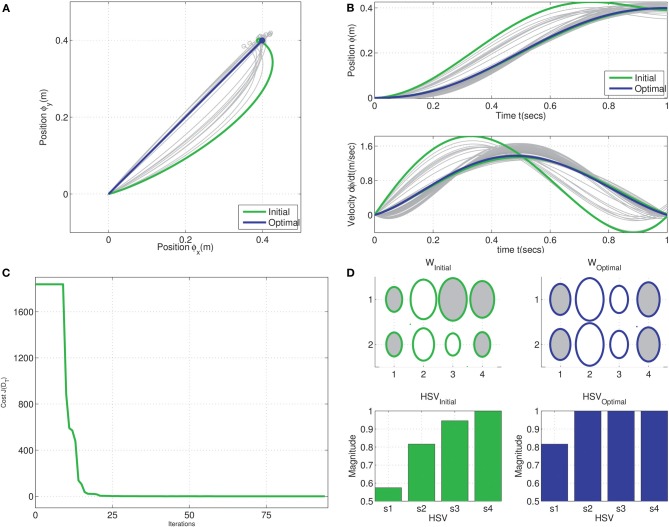
**Generalization of the minimum dimensional control for reaching tasks in the tethered mass system**. Optimization was initialized with a trajectory passing through a via-point offset by 0.075 m from the straight line connecting origin and target ϕ_*d*_ = (0.4, 0.4); Gradual convergence to Cartesian straight-lines with bell-shaped time-normalized velocity profiles seen during intermediate stages of the optimization (shades of gray) in the, **(A)** Cartesian endpoint trajectories, and **(B)** position and velocity traces of endpoints. **(C)** Change in *J*(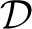
_*T*_) cost with each iteration of optimization, and **(D)** Hinton diagram of the initial and optimal weights and the corresponding normalized Hankel singular values.

The generalization of the MDC is demonstrated in Figure 7. The numerical optimization was initialized with a trajectory passing through a via-point located at (0.4, 0.3). The MDC optimization converged toward the straight line trajectory with a bell-shaped velocity profile as seen in Figure 7B. The change in cost with each iteration of optimization shows that the algorithm rapidly converges towards the optimal solution of cost *J*(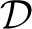
_*T*_). The synergy weight matrix in the optimal case consists of identical values in each row indicating that the MDC solution yields identical force inputs to the system for the reaching task.

### 3.3. TSDA on the kinematic chain

In case of the non-linear compliant kinematic chain system, the empirical balancing procedure was used to compute TSDA. Again a set of four benchmark trajectories *T*_1 … 4_ were utilized. In each case, the arm was initialized with the angles θ(*t*_0_) = [−π/16, π/8]^*T*^, i.e., the rest position. Similar to the linear system experiments, each trajectory described a motion from the initial position to an end position [0.5, 0.2] in the Cartesian space. Again, the trajectories were obtained by fitting cubic splines to Cartesian via-points with smoothness conditions enforced at the boundaries (2nd order boundary conditions set to 0), each representing a variation on the reaching task. Inverse kinematics was then used to compute the joint angle trajectories for each trajectory; the “*down*” configuration was utilized mimicking the reaching behaviors in humans. The required torque τ_*i*_(*t*) = [τ_*i*_1__(*t*), τ_*i*_1__(*t*)]^*T*^ corresponding to each task *T*_*i*_ was then computed by using the inverse dynamics of the system. The weight matrix was then computed for each trajectory using a least-squares procedure. For the experiments carried out, analysis was restricted to the Legendre polynomial synergies since it offered a better fit of the desired torques with a relatively low order in comparison with the Fourier basis synergies.

**Figure 8:**
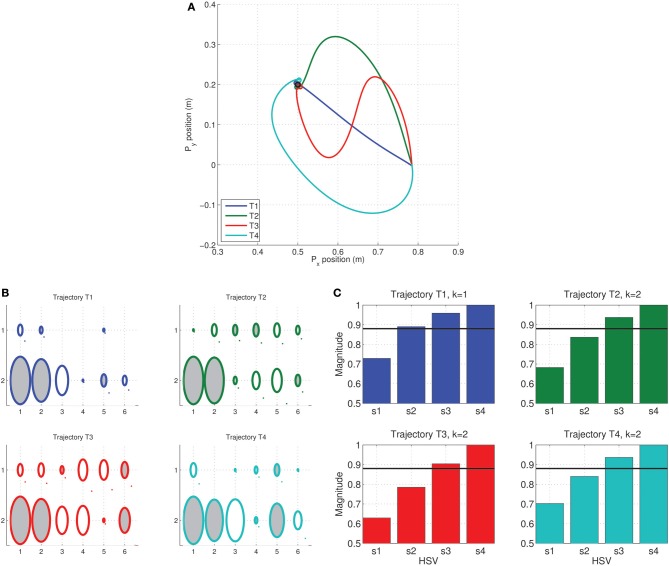
**Trajectory Specific Dimensionality Analysis (TSDA) computed on Legendre basis synergies (order 7) actuating the compliant kinematic chain system, the task was to reach position *P*_*d*_ = (0.5, 0.2) in a time span of 2.5 s, the initial condition was a nearly fully extended kinematic chain. (A)** Four benchmark trajectories [*T*_1_, … *T*_4_] traced by the mass under synergy control—synergy weights were computed from via-points using a least-squares approach; **(B)** Hinton diagram of the weight matrix (ellipse size is the magnitude, a dark region denotes positive weight and white region denotes a negative weight). **(C)** The normalized empirical HSV magnitudes for the non-linear reformulated composite systems for each trajectory. For a threshold magnitude choice of *t*_*r*_ = 0.935, represented by the solid black line, the DR was computed as 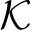 = [1, 2, 2, 2]. The straight line trajectory *T*_1_ has minimum dimensionality as measured by the HSV magnitudes.

The endpoint trajectories for the four cases using Legendre basis synergy control is seen in Figure 8A. The weight matrix is represented by the Hinton diagram in Figure 8B. From the size of the shaded ellipse, it can be seen that in all four cases, the contribution of the proximal joint inputs is much higher. The temporal aspects of the trajectories can been seen in the relative contributions of the negative weights (ellipses with white shading). Again, the corresponding constrained reformulation was obtained and the empirical balancing procedure was utilized to compute the approximate HSVs. Since the Legendre polynomial synergy magnitudes are bounded, the empirical Gramians were computed from the state trajectories resulting from applying unit impulses across the inputs of the constrained reformulated system.

The application of empirical balancing in this framework is equivalent to activating combinations of the synergies with bounded impulses; the magnitudes were chosen from a uniform distribution about an input ball of same dimension as the number of synergies, i.e., of dimension *S*. The HSVs corresponding to each task *T*_*i*_ computed by this method can be seen in Figure 8C. The DR using a threshold choice of *tr* = 0.935 was obtained as 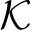 = [1, 2, 2, 2]. Similar to the earlier linear example, it can be observed that the straight line trajectory with a sigmoidal profile seemingly has the minimum dimensionality of 1. This observation was examined in detail in the MDC experiments, presented next.

### 3.4. Minimum dimensional control in kinematic chain

The MDC experiment was repeated on the kinematic chain system for a set of reaching targets within the workspace of the arm. Similar to the linear case, the minimization process was initiated with the constraints of zero velocity enforced at the boundaries. A constraint tolerance of ϵ = 10^−2^ was used as a terminal criterion for the minimization.

**Figure 9:**
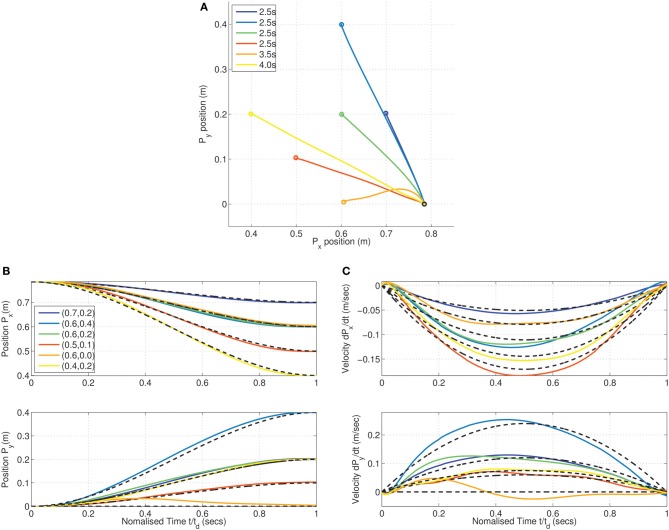
**Minimum dimensional control on the kinematic chain for reaching various positions using Legendre basis synergy (order 7)**. Minimum dimensional trajectories were obtained for targets (0.7, 0.2), (0.6, 0.4), (0.6, 0.2), and (0.5, 0.1) in a duration of 2.5, (0.6, 0.0) in 3.5 s and (0.4, 0.2) in 4 s, respectively. **(A)** Near straight lines seen in the Cartesian endpoint trajectories. **(B)** Trajectory of endpoint is sigmoidal, and **(C)** time-normalized velocity profiles show slightly skewed bell shapes. The peaks of the velocity profiles, however, are close match to the minimum acceleration (MA) criterion result.

The (locally) optimal trajectories resulting from MDC can be seen in Figure 9 for the Legendre basis synergies. Smooth sigmoidal near-straight line trajectories emerge for some movement durations; the results were obtained for different movement durations of *t*_*d*_ = 2.5, 3.5, and 4 s. In contrast with the linear MDC case minor skewing effects can be seen in the velocity profiles. These effects are a consequence of the approximate fitting offered by a fixed set of synergies in order to meet the terminal boundary conditions.

Similar to the linear system experiments, the peak velocity obtained for the reaching movements is dependent on the movement amplitude. It can also be seen in this case that the correspondence of the obtained trajectories to the Minimum Acceleration (MA) model (Ben-Itzhak and Karniel, 2008) is greater (black dashed lines in Figures 9A,B).

Clearly, a close correspondence is seen between the obtained reaching trajectories and human reaching behavior as reported by Morasso (1981) and by several others.

**Figure 10:**
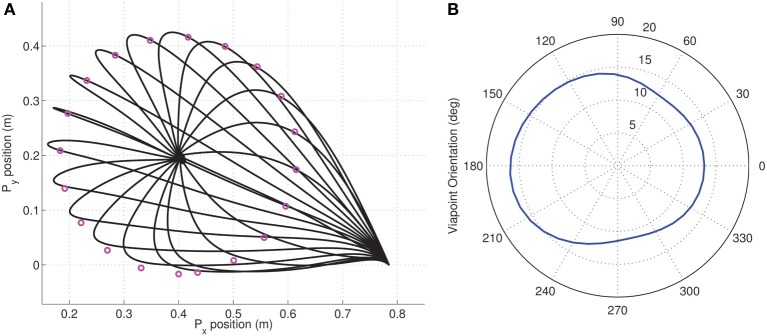
**Dimensionality analysis for via-point tasks. (A)** A set of cartesian via-points were specified on a circle of radius 0.216 m centered on the target (0.4, 0.2) for a reaching movement from the initial position; **(B)** Polar plot of the variation in the dimensionality performance index against orientation of via-point with respect to origin. The minimum index of 11.21 is located at the orientation of 286.15° corresponding to the via-point at (0.61, 0.14) which is very close to the straight line linking origin and target.

As in the earlier linear system experiments, we use the MDC framework to analyze the reduction in dimensionality in via-point tasks. Via-points are chosen to lie on a circle about the target position (as seen in Figure 10). Again, the via-points are specified to be reached at exactly at half of the movement duration. For each trajectory, the appropriate synergy weight matrix was computed. The variation of the dimensionality performance index with respect to via-point orientation is obtained, as seen in the polar plot in Figure 10B.

In contrast with the linear example, it can be seen that there exists a non-zero minimum value of the performance index. The reaching target of (0.4, 0.2) was chosen from the set of points investigated in the earlier MDC reaching experiments. For this target position, it can be seen that the via-point resulting in the best DR lies on the straight line connecting origin and the target position. However, reversal tasks are greater in dimensionality implying that they are more complex to achieve in the kinematic chain system.

**Figure 11:**
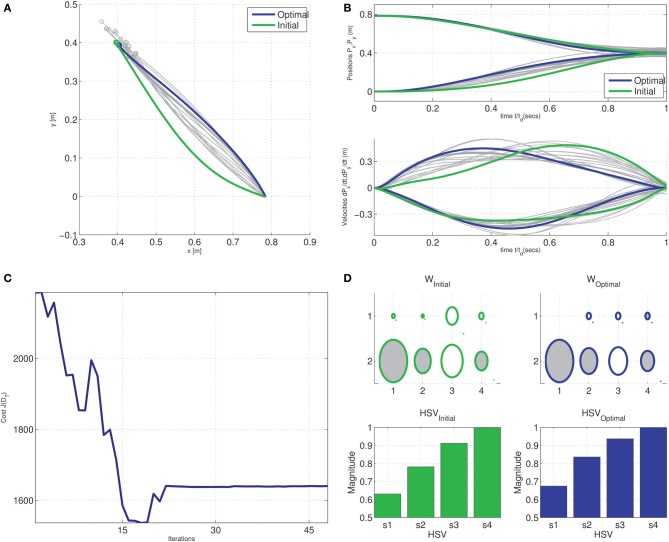
**Generalization of the minimum dimensional control in reaching in the compliant kinematic chain system**. Optimization was initialized with a trajectory passing through a via-point offset by 0.04 m from the straight line connecting origin and target (0.4, 0.4); Gradual convergence to Cartesian straight-lines with bell-shaped time-normalized velocity profiles seen during intermediate stages of the optimization (shades of gray) in the, **(A)** cartesian endpoint trajectories, and **(B)** position and velocity traces of endpoints. **(C)** Change in *J*(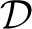
_*T*_) cost with each iteration of optimization, and **(D)** Hinton diagram of the initial and optimal weights and the corresponding normalized Hankel singular values.

The generalization of the MDC in the non-linear case can be seen in Figure 11. The numerical optimization is initialized with a trajectory passing through a via-point located at (0.6, 0.1). The MDC converges toward a trajectory close to the straight line with bell-shaped velocity profile as seen in Figure 11B. The change in cost with each iteration of optimization shows that the algorithm rapidly converges towards the optimal solution of cost *J*(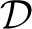
_*T*_). In contrast with the linear result earlier, at some stages of the optimization, the intermediate cost is below the terminal cost as seen in Figure 11C. This is a consequence of the *active-set* algorithm which results intermediate solutions which do not obey the constraints. The convergent (locally) optimal solution obeys the terminal position and velocity constraints as seen in Figure 11B.

## 4. Discussion

In this paper, we develop a quantification for the reduction in the behavioral dimensionality in a system due to control in the form of muscle synergies. When using the temporal synergy formulation, the behavior dynamics are dependent on the synergy basis and the weight matrix. We model this as a trajectory-specific constrained reformulation of the dynamics of the system. Using the approach of system balancing, we quantified the reduction in dimensionality using a threshold-normalized Hankel Singular Value (HSV) measure this process computes the dimensionality of the subspace of the dynamics of the balanced system. Using our method of Trajectory Specific Dimensionality Analysis (TSDA) we show that various trajectories that satisfy task constraints can be compared in terms of reduction in dimensionality in a system and synergy basis specific manner. We then develop a method for minimization of this dimensionality in our model of Minimum Dimensional Control (MDC). The method yields the weight matrix corresponding to the minimum dimensional trajectory that satisfies task constraints using a constrained minimization of the HSV measure. The proposed methods were simulated on biologically-relevant linear (tethered mass) and non-linear (compliant kinematic chain) systems. Using idealized temporal synergies, a task, synergy, and system specific reduction of dimensionality of behavior due to control using muscle synergies was demonstrated. The trajectories obtained as a consequence of this minimization, closely correspond with observations of some of the kinematically invariant features in human movements. We therefore propose that a dimensionality reduction principle might underlie motor control as a direct consequence of developmental necessities.

Bernstein's “*degrees of freedom problem*” remains a seminal observation of natural motor coordination, and continues to challenge our biological understanding as well presenting a fundamental obstacle to biomimetic engineering. Some kind of DR surely occurs, but whether it is an implicit/ emergent phenomenon (e.g., Lagrangian optimization), or an explicit ‘simplifying’ evolutionary and/or developmental strategy remains a conundrum. The muscle synergy hypothesis suggests that the DR is a fundamental advantage resulting from the partitioning of the space of inputs (Alessandro et al., 2013). However, it has faced criticism. Although statistical regularities seem to be present in the measurements of EMG, and kinematic data from subjects performing behavioral tasks, the extracted synergies are strongly dependent on the nature of observations that can be made (Steele et al., 2013). Despite recent approaches for careful experiment design have aimed at addressing this criticism, the perception that this hypothesis represents only a phenomenological view of motor control seems hard to shake off (Tresch and Jarc, 2009). Falsification of this theory requires careful identification of the actual functionality offered by muscle synergies toward learning and control of optimal motor behavior.

Our view is that for DR to exist in biological organisms, it would need to impact on the organism's behavior, as this is a major determinant of fitness. Muscle synergies would probably only evolve if they had a positive influence on an organisms ability to solve tasks, learn motor skills, and adapt to changes. To this end, TSDA quantifies the DR in dynamic behavior. The dimensionality of behavior is taken to denote the dimensionality of the state-space of the system under synergy control. It is specific to a task and to a defined set of synergies. The dynamic models obtained through the task-specific reduction of this state-space are reminiscent of the internal model hypothesis (Wolpert et al., 1998; Kawato, 1999). Although we do not investigate this relationship further in this work, the task-specific reduced internal representations associated with our MDC trajectories could be very relevant for motor planning for tasks (Braun et al., 2009). Through following these minimum dimensional trajectories, an organism could minimize the neural complexity required for learning internal models.

Attempts have been made to fit synergy data extracted from behavior onto musculoskeletal models (Neptune et al., 2009; McKay and Ting, 2012; Steele et al., 2013). Our approach could potentially complement this analysis and allow the quantification of the differences between synergies extracted by various methods on a given dataset. This would then be a synthetic approach for testing the validity of any set of synergies toward simplifying the control and learning problem. Although we only employed fictitious synergies composed of idealized bases of Legendre and Fourier components, our methods can be applied to any synergy set specified by a time series. TSDA can also potentially be used to test the validity of a task definition, in terms of constraints presented to subjects, as well as the nature and quality of the number of EMG measurements that are employed for synergy extraction. Although our demonstration focussed on the temporal synergy model, in principle the methods can be used for quantification of other models of synergies such as the time-varying synergies (d'Avella and Bizzi, 2005).

The methods we developed in this paper represent a control-theoretic perspective on the muscle synergy hypothesis. This entails a synthetic examination of the role of muscle synergies in acting as facilitators of optimization through control dimensionality reduction. In this view, it is not only important to extract spatio-temporal regularities from biological behavior datasets, but also to carefully examine if task control and learning is indeed facilitated (Alessandro et al., 2013; de Rugy et al., 2013). In particular, Berniker et al. (2009) suggested that synergies represent a task-variable specific reduction in controller dimensionality. We essentially extend this view by quantifying a task-variable and synergy basis specific reduction—thereby allowing us to understand the temporal aspects of motor behaviors. Our approach is also closely related to a recent analysis of the synergy hypothesis from an intermittent hierarchical control perspective (Karniel, 2013). In principle, the notion of minimal intermittancy and our concept of minimum dimensionality both have an underlying objective of minimizing control effort, and further investigation of this relationship is definitely warranted.

The methods presented in this paper also have potential applications in the control of artificial systems such as robots. Current state-of-art methods such as policy gradients (Peters and Schaal, 2008), and the *PI*^2^ algorithm (Policy Improvement through Path Integrals) (Theodorou et al., 2010) have been used for demonstrations of reinforcement learning being applied to high-dimensional robot systems. In comparison with model-free reinforcement learning, model-based methods offer several advantages such as the ability to update policies offline and then performing sporadic updates from real-world data. Also model-based methods allow safe exploration without risking damage of robots. Our approach naturally facilitates tractable model based learning and could serve as a planning tool acting in concurrence with existing reinforcement algorithms in order to speed-up learning.

In several reinforcement learning proposals, the trade-off between exploration and exploitation is often discussed. It is important to note that a method based on reduced dimensional internal models, although offering potential speed-up of learning, could also limit the scope of the obtained solutions—i.e., the learning could converge to suboptimal behavior. Within the context of our framework, we believe that this problem could instead be tackled by a developmental scheme of progressively increasing internal model dimensionality along with the acquisition of control of newer skills. This notion is similar to the developmental hypothesis of degree-of-freedom freeing and unfreezing (Bernstein, 1967). Consequently, the developmental increase in the number of synergies to cope with increased task requirements (Dominici et al., 2011) would than be equally supplemented by a progressive increase in internal model dimensionality. Thus task-specific models of internal models of increasing complexity would progressively be evaluated as the organism matures.

Although the scope of this paper was limited to the analysis of deterministic continuous-time systems, the methods can in principle be adapted to deal with stochastic effects and discretization. The resulting approach could then be used to supplement existing state-of-art methods in iterative stochastic optimal control (Theodorou et al., 2010). Furthermore, although the investigations focussed on a feedforward control scenario, the methods can easily incorporate a feedback control formulation of plant dynamics; the models we tested already include a weak mechanical feedback in the form of passive joint compliance. Nevertheless, it must be noted that several existing models in the synergy hypothesis suggest that muscle synergies are a high-level feedforward control scheme that incorporates low-level feedback (d'Avella et al., 2003; Hart and Giszter, 2004; Ivanenko et al., 2004; Ting and Macpherson, 2005; Tresch et al., 2006). In an artificial context, this notion has also been explored in the design of dynamical movement primitives (Ijspeert et al., 2013) wherein the policies encode trajectory features while the primitives themselves can then be modified online in a smooth manner taking into account disturbances etc. due to their dynamic nature.

The Optimal Control Theory (OCT) models of human motor behavior originate from a evolutionary perspective; there is a fitness-driven necessity for behaviors to be optimal. Various Lagrangians have been proposed to quantify task optimality depending on the different perspectives of the system such as the output (kinematic) (Flash and Hogan, 1985), control input (minimum variance Harris and Wolpert, 1998, minimum norm Dean et al., 1999), or intermediate variables (minimum torque Nakano et al., 1999). However, it must be noted that OCT hypotheses employ relatively complex mathematical techniques; current theoretical limitations mean that OCT methods can only be applied analytically on relatively simpler models such as linearized models of the oculomotor system or limb movements (Harris and Wolpert, 1998). Also, there is no testable suggestion so far as to how and where the optimization might actually be happening in terms of actual neural mechanisms. The method proposed in this paper is possibly a step toward this goal, since we relate optimization to the actual recruitment of synergies to accomplish tasks.

From a developmental perspective, the process of acquisition of motor coordination is gradual and seemingly composed of intermediate stages of learning (Sporns and Edelman, 1993). If we consider that optimal solutions exist in a high dimensional space (system dynamics, neural control input) unique to an individual organism, then fitness must also depend on the ability to find good solutions in the developmental time frame (Harris, 2011). Searching for an optimal trajectory has a little value if it takes a long time to find. We propose that the time taken to learn an optimal control, which we call “learnability” is itself an important parameter in a self-organizing system (Kuppuswamy et al., 2012). DR is one possibility which may speed up learning, but there might be a trade-off with precision and learning rate to the extent that non-redundant degrees of freedom are eliminated. Our approach provides a mechanism to examine this hypothesis through the measurement of dimensionality of empirically measured trajectories relative to some assumed or computed basis set of synergies.

The most interesting results obtained through our methods are the smooth straight-line sigmoidal trajectories with bell-shaped velocity profiles as the minimum dimensional solution to reaching tasks. The similarity at the output for two basis sets (Legendre and Fourier) and for both linear and non-linear systems suggests the possibility of some kind of invariance at the output task variable level. We also observed that the symmetry of the velocity profiles is strongly affected by the specification of boundary conditions on the behaviors. Smoothness implies a potential relationship between DR and bandwidth reduction. Clearly, task demands place constraints on possible trajectories, and hence on their spectral content. In point-to-point reaching trajectories with zero velocity boundary conditions, the temporal truncation forces a strictly infinite bandwidth, with rapidly decaying spectral energy limiting envelope (Harris, 2004). The fastest movement that can be achieved without exceeding this spectral limit are the family of minimum square derivative functions, such as minimum acceleration for 2^nd^ order systems, or minimum jerk for 3^rd^ order systems. The DR trajectories had lower peak velocities than expected from the minim jerk profile, but were similar to minimum acceleration (dotted lines in Figures 5, 9). The relationship between DR and low bandwidth is unclear at present, but has two important implications.

If this invariance is upheld, it implies that the choice of basis set is not critical (presumably provided the output trajectory can be spanned by the input basis set). Indeed, it may reflect the possibility that DR occurs at the output directly. In our work we only examine the state-space dimensionality and the computation of minimum dimensional weight matrix. In principle, this approach may also be used for investigating the optimal temporal characteristics of the basis set themselves. For example, using the Legendre polynomial basis, we observe a reduction in dimensionality across tasks, both in the input as well as in the output. In this respect, it is interesting that low bandwidth signals also have low Shannon numbers (although the Shannon number is an imprecise measure of signal dimension when duration is finite).

Second, there is a coincidence between low dimensionality and optimal control. That is, if low dimensionality is maintained, optimal or near-optimal trajectories are automatically generated for a given set of boundary conditions, and the curse of dimensionality is largely circumvented. An alternative is that the optimality approach itself is a misconstrued attempt to explain low dimensionality via a Lagrangian. However, for the minimum variance model, it would be difficult to explain the known presence of signal-dependent noise unless the noise is somehow a product/compensation for DR.

This last point is also relevant to synthetic (robotic) systems. Minimization of biologically relevant Lagrangians in synthetic systems does not necessarily lead to biologically realistic behavior, but depends on the synthetic architecture. For example, minimizing reaching time in a natural system appears to be achieved by the smooth bell-shape velocity profiles, but in a linear robot the same Lagrangian (functional mimicry) would be optimized by bang-bang control leading to skewed velocity profiles. In any case, finding such solutions in real-time is non-trivial, and often natural behavior must be programmed explicitly into the artificial system (esthetic mimicry) (Harris, 2009). However, when we consider DR as the underlying principle for generating natural behavior, we envision that functional mimicry in a robot would produce similar or the same natural behavior. It is not entirely clear at present, how precisely the mimicry would need to be. It is plausible that only crude approximations are needed. Furthermore, although we investigated two relatively simple systems performing reaching and via-point type tasks, the methods are computationally applicable to any control-affine systems. Thus in principle, these methods could be used to compute “natural” behaviors in robots of a variety of morphologies. A related application would be to optimize behavior in artificial systems that are driven by pattern based mechanisms such as Central Pattern Generators (CPG) (Ijspeert, 2008). Our approach is thus a potential path toward robots with neurally inspired motor control of reduced complexity.

## Funding

This research was supported by the European Community under the 7th Framework Programme by the projects RobotDoc (Grant Agreement No. 235065), a Marie Curie Action ITN, and AMARSi (Grant Agreement No. 248311).

### Conflict of interest statement

The authors declare that the research was conducted in the absence of any commercial or financial relationships that could be construed as a potential conflict of interest.
